# Pre- or post-chemotherapy: effect on PSMA uptake

**DOI:** 10.1186/s13550-025-01229-3

**Published:** 2025-04-07

**Authors:** Surekha Yadav, Abuzar Moradi Tuchayi, Moein Moradpour, Fei Jiang, Roxanna Juarez, Ivan de Kouchkovsky, Robert R. Flavell, Rahul R. Aggarwal, Thomas A. Hope

**Affiliations:** 1 Department of Radiology and Biomedical Imaging, University of California San Francisco, San Francisco, CA USA; 2 Department of Epidemiology & Biostatistics, University of California, San Francisco, USA; 3 Department of Medicine, Division of Medical Oncology, University of California, San Francisco, CA USA; 4 Helen Diller Family Comprehensive Cancer Center, University of California, San Francisco, CA USA; 5 Department of Radiology, San Francisco VA Medical Center, San Francisco, CA USA; 6 Department of Radiology and Biomedical Imaging, University of California, San Francisco, 185 Berry Street, Lobby 6, Suite 350, San Francisco, CA 94107 USA

## Introduction

Despite multiple therapies that delay disease progression and prolong life, metastatic castration-resistant prostate cancer (mCRPC) remains the second leading cause of cancer related death in American men [1, 2]. Radioligand therapy (RLT) targeting Prostate Specific Membrane Antigen (PSMA) has been shown to improve clinical outcomes and have a favorable safety profile [3–7]. In the Phase 3 VISION study, ^177^Lu-PSMA-617 (vipivotide, Pluvicto, Novartis) was shown to prolong overall survival (OS) and improve quality of life in patients with mCRPC relative to best supportive care [5, 7], while the Phase 2 TheraP Trial demonstrated that ^177^Lu-PSMA-617 resulted in a higher rate of PSA decline relative to cabazitaxel chemotherapy [6].

More recently, PSMAFore studied the role of ^177^Lu-PSMA-617 prior to chemotherapy in mCRPC patients compared to androgen receptor pathway inhibitor (ARPI) switch, and demonstrated that ^177^Lu-PSMA-617 prolongs radiographic progression-free survival [8, 9]. There are two additional ongoing Phase 3 studies evaluating the benefit of PSMA RLT prior to chemotherapy: SPLASH (NCT04647526) and ECLIPSE (NCT05204927).

Elevated PSMA expression has been correlated with improved response to PSMA RLT [10]. Conversely, patients exhibiting low PSMA expression or discordant metastases generally demonstrate suboptimal responses [11]. The change in PSMA expression with different therapies may impact the timing when one would prefer to use PSMA RLT. It is unknown how taxane-based chemotherapy impacts PSMA expression, and therefore it is not clear in what setting PSMA expression is highest. In this study, we aimed to measure the impact of taxane-based chemotherapy on PSMA uptake.

## Materials and methods

### Study population

We conducted a retrospective study of all patients imaged before and after a minimum of two cycles of taxane-based chemotherapy (including cabazitaxel/docetaxel alone or with platinum based chemotherapy) at our institution between June 2021 and Apr 2024, who had a pre-chemotherapy PSMA PET performed within six months prior to the first cycle and post-chemotherapy PET before initiation of subsequent therapy. Patients who were administered ARPIs simultaneously or with a history of prior taxane based therapy in a mCRPC setting were excluded from the study. The institutional review board approved this retrospective study and the requirement to obtain informed consent was waived.

### PSA response to chemotherapy

Serum PSA levels served as the standard of reference for response assessment to chemotherapy. Change in serum PSA from the day of first cycle to the lowest value prior to subsequent therapy was used to calculate PSA response. Response to chemotherapy was defined as a PSA decline of 50% from baseline. PSA progression was defined as a PSA increase of more than 25% from baseline of at least 2 ng/ml above nadir while on chemotherapy per the criteria of the Prostate Cancer Clinical Trials Working Group 3 [12]. The remainder of patients were classified as having stable disease. For the purposes of analyzing the changes in quantitative PET parameters, patients with PSA progression and those with stable disease were grouped together as non-responders and compared against the responders.

### PSMA PET scan analysis

PSMA PET imaging was performed using ^68^Ga-PSMA-11 (gozotetide, Illucix/Telix; Locametz/Novartis; UCSF) or ^18^F-DCFPyL (piflufolostat, Pylarify, Lantheus). Scans were analyzed using MIM PET diagnostic visualization software (MIM Software, Cleveland, OH, USA). Two thresholds for lesion segmentations were used: (1) SUVmean of 3 for osseous lesions, lymph nodes and extrahepatic soft tissue lesions and (2) SUVmean of liver x 1.5 for liver lesions. Physiological areas of uptake were manually removed to obtain PSMA avid lesions for osseous, lymph node, and soft tissue lesions. Four parameters were calculated for each region: maximum standardized uptake value (SUVmax), mean SUV (SUVmean), total tumor volume (TTV, which is the summed tumor volume of the segmented PSMA avid lesions), and total lesion PSMA uptake (TL-PSMA, which is TTV x SUVmean). The ratio of PSA levels at the time of imaging to TL-PSMA (PSA/TL-PSMA) was calculated at both baseline and post-treatment PET. Changes in total tumor volume (TTV) were quantified, and chemotherapy response was assessed according to the Response Evaluation Criteria in PSMA PET/CT (RECIP 1.0) [13], combining changes in TTV with the occurrence of new lesions to categorize response as RECIP CR (complete response), PR (partial response), SD (stable disease) and PD (progressive disease).

### Statistical analysis

Descriptive statistics were used to describe quantitative variables. Categorical variables were reported as counts and percentages. A Student’s t-tests was used to compare the changes in quantitative parameters post-treatment and between responders and non-responders, as well as to compare changes in SUVmean and PSA response between patients with baseline SUVmean ≥ 10 and those with baseline SUVmean < 10. A p-value < 0.05 was considered significant. Pearson correlation of PSA/TL-PSMA before and after chemotherapy was analyzed. Additionally, the maximum decline in PSA after taxane-based chemotherapy was reported for each patient using waterfall plots.

## Results

### Patient characteristics

In total, 42 patients were analyzed (Table 1). Baseline PSMA PET was performed within a median of 52 days (IQR, 31–107 days) before the first cycle of taxane-based chemotherapy, and the post-treatment scan was conducted within a median of 34 days (IQR, 19–68 days) after the last cycle.

**Table 1:** Patient demographics and clinical characteristics

Patients *N* = 42	
Age median, median (IQR)	74.5 (70.5–79)
Gleason grade group median, median (IQR)	4 (3–5)
Radical prostatectomy, n (%)	13 (31)
Radiation therapy, n (%)	38 (90)
Prior ARPI, n (%)	42 (100)
Prior ADT, n (%)	42 (100)
Taxane chemotherapy, n (%)	
Docetaxel, n (%)	33 (78)
Cabazitaxel, n (%)	9 (22)
Other (Immunotherapy/platinum-based), n (%)	12 (29)
PSA prior to chemotherapy, median (IQR)	66.4 (20.1–159.2))
PSA post chemotherapy, median (IQR)	48.0 (17.7–195.3)
Number of taxane-based chemotherapy cycles *	
PSA responders, median (IQR)	7.5 (5.7–8.5)
PSA non-responders, median (IQR)	4 (3–6)
Location of disease, n (%)	
Bone	40 (95)
Lymph node	29 (69)
Visceral	22 (52)
Time to imaging in days, median (IQR)	
PSMA PET prior to chemotherapy	52 (31–107)
PSMA PET post chemotherapy	34 (19–68)

* p value < 0.001

### PSA response to taxane-based chemotherapy

**Fig. 1:**
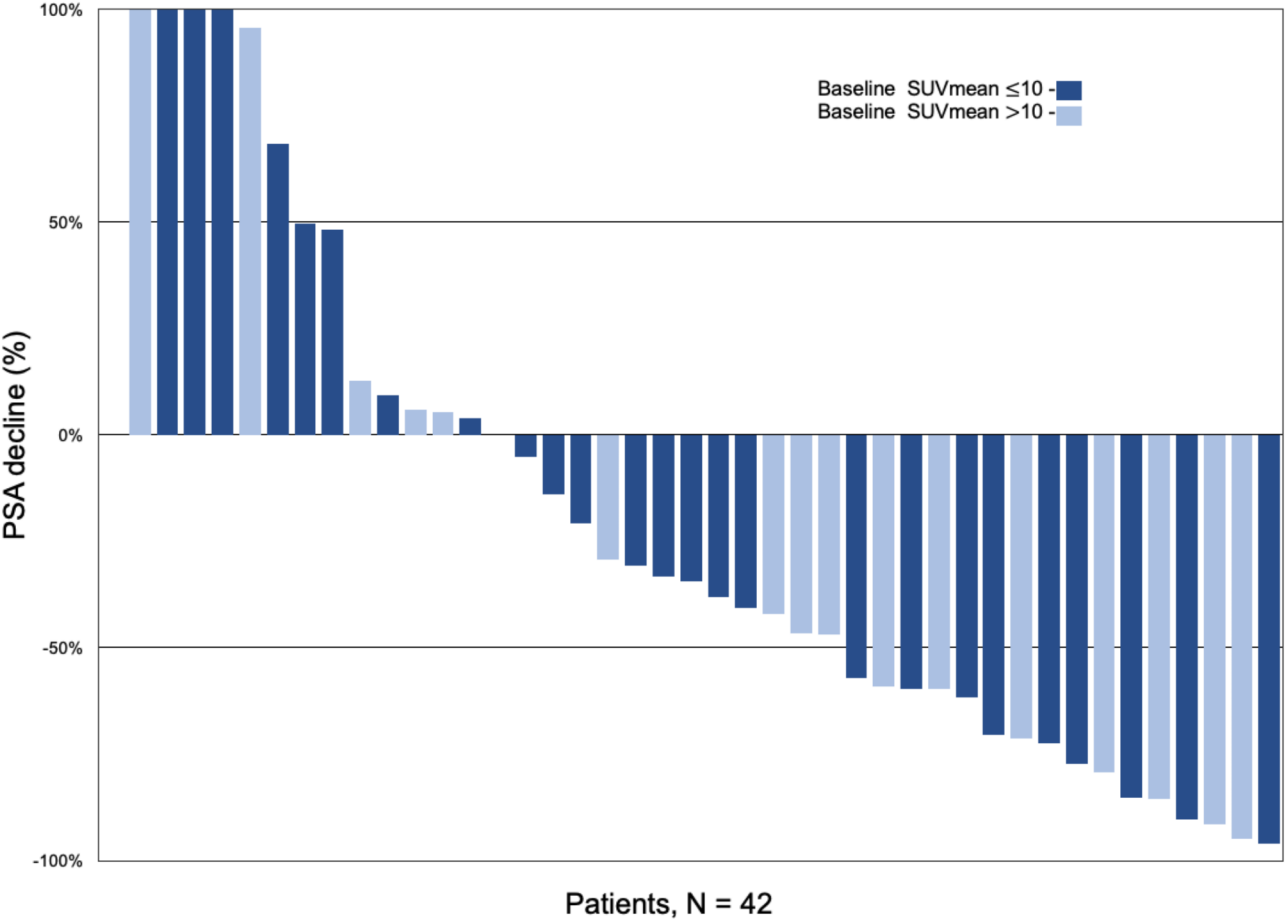
Waterfall plot showing the change in PSA in response to taxane-based chemotherapy. 38% achieved a 50% reduction in PSA, stratified by baseline SUVmean on PSMA PET

Patients received a median of 5 (range, 3–7) cycles of taxane-based chemotherapy. The median PSA change after chemotherapy was −36% (IQR, −68% to + 6%). Of the 42 patients, 16 (38%) were responders (i.e. had a 50% drop in PSA), with a median PSA decrease of 75% (IQR, −87% to −61%). 26 (62%) patients were non responders, with a median PSA increase of 2% (IQR, −33–49%) (Fig. 1). Among the non-responders, 18 patients (43%) achieved stable disease and 8 patients (19%) had PSA progression.

### Quantitative PSMA PET parameters

For the 42 patients analyzed, the pre-chemotherapy SUVmean, SUVmax, TTV and TL-PSMA were 9.5 (IQR, 7.2–12.8), 45.3 (IQR, 32.2–78.3), 390.0 cm³ (IQR, 115.2–910.7 cm³) and 4241.6 cm³ (IQR, 873.1–7813.6 cm³). The post-chemotherapy SUVmean, SUVmax, TTV and TL-PSMA were 8.5 (IQR, 6.4–10.5), 49.0 (IQR, 32.7–78.0), 833.7 cm³ (IQR, 213.4–1410.1 cm³) and 6411.8 cm³ (IQR, 1278.9–10790.5 cm³). The median percentage changes in SUVmean, SUVmax, TTV and TL-PSMA were −8% (IQR, −29% to + 6%; *p* = 0.11), + 0.003% (IQR, −35% to + 32%, *p* = 0.72), + 47% (IQR, −6% to + 100%, *p* = 0.09), and + 38% (IQR, −23% to + 100%, *p* = 0.31).

Of the 42 patients, response according to RECIP criteria showed that 8 patients (19%) achieved partial response, 27 patients (64%) had progressive disease, and 7 patients (17%) maintained stable disease. Additionally, 57% of patients showed decrease in SUVmean, out of which 7 patients had their SUVmean drop below 10 after chemotherapy.

**Fig. 2:**
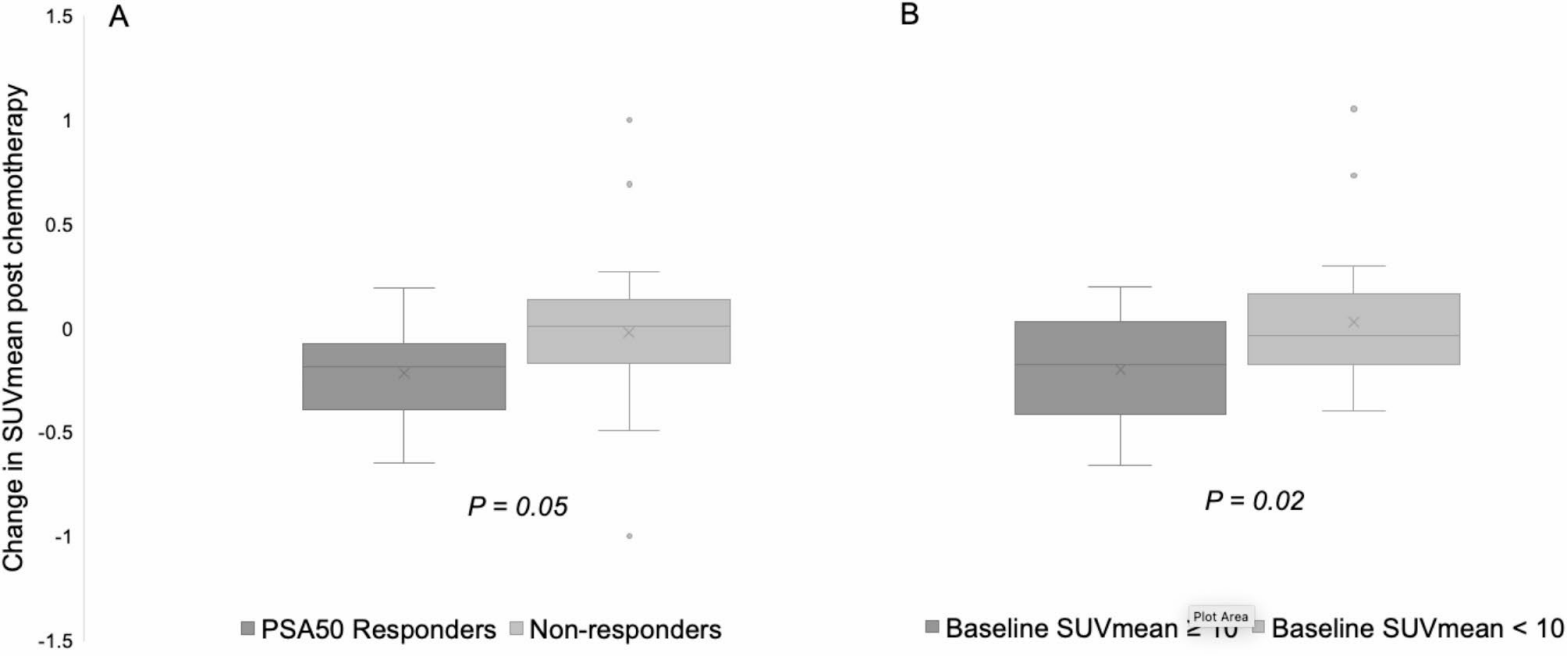
Box plots showing the change in post-treatment PSMA PET SUVmean from baseline between PSA50 responders and non-responders (**A**) and between those with baseline SUVmean ≥ 10 and < 10 (**B**)

Patients with an SUVmean ≥ 10 at baseline had a larger decrease in SUVmean post-treatment (baseline SUVmean ≥ 10, SUVmean fell by −18% [IQR, −41% to + 2%]; versus, baseline SUVmean < 10, SUVmean fell by − 6% [IQR, −18% to + 15%], *p* = 0.02; Fig. 2). Additionally, patients with a higher SUVmean had a higher PSA response on average, however this was not statisticall significant (baseline SUVmean < 10, PSA decreased by 31% [IQR, −60% to + 9%]; versus baseline SUVmean ≥ 10, PSA decreased by 42% [IQR, −72% to + 5%]; *p* = 0.22).

### PSMA expression and effect of chemotherapy

**Fig. 3:**
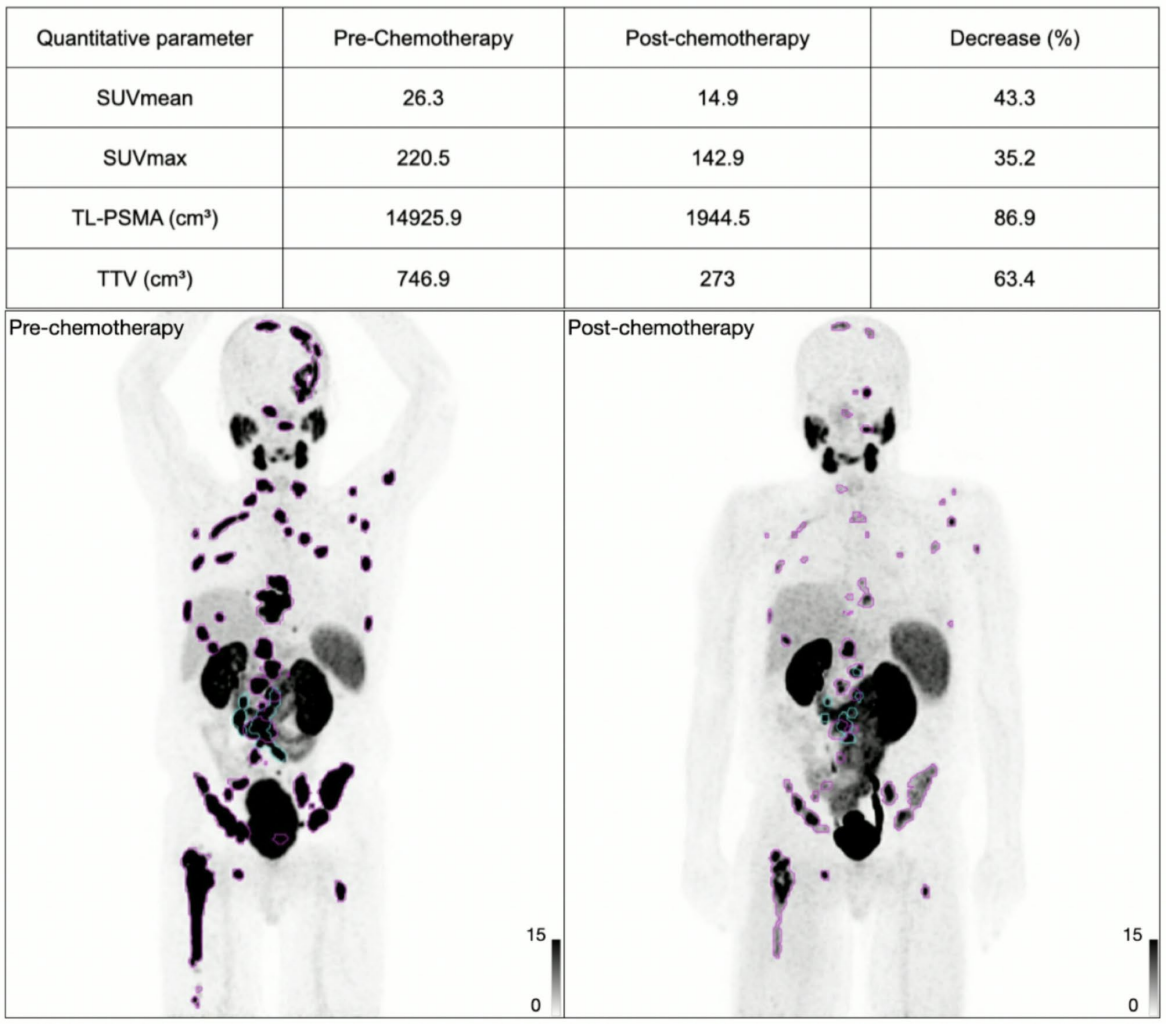
A 75-year-old man with mCRPC, demonstrating a decrease in PSMA expression after 7 cycles of docetaxel. The patient had a PSA decline of 95% and a 43% decrease in SUVmean

Among the PSA responders, the median percentage changes in SUVmean, SUVmax, TTV and TL-PSMA were −19% (IQR, −39% to −7%), 20% (IQR, −37% to + 13%), + 14% (IQR, −47% to + 100%) and 2% (IQR, −65% to + 100%). Among the PSA non-responders, the median percentage changes in SUVmean, SUVmax, TTV and TL-PSMA were + 1% (IQR, −17% to + 14%), + 13% (IQR, −28% to + 54%, and *p* = 0.17), + 51% (IQR, −2% to + 100%, *p* = 0.21) and + 42% (IQR, + 1% to + 100%, *p* = 0.19; as shown in Supplemental Fig. 1). The decrease in SUVmean was greater in PSA responders compared to non-responders (*p* = 0.05, Figs. 2 and 3).

Additionally, there was a positive correlation of 0.60 (*p* < 0.001) between the PSA/TL-PSMA ratio on the baseline and post-treatment PET (Supplemental Fig. 2). 27 (64%) patients had a greater than 50% difference in their PSA/TL-PSMA ratio after treatment compared to baseline. This variation resulted in a difference in response classification, with 25 patients showing discordant results when assessed using PSA response per PCWG3 versus PSMA PET response per RECIP 1.0. Notably, 8 patients classified as PSA responders by PCWG3 criteria were classified as PD according to RECIP 1.0 criteria.

## Discussion

In this study, we demonstrate that PSMA uptake is reduced after taxane-based chemotherapy, which was significant in patients with high baseline SUVmean and responders. Additionally there was an increase in the TTV and TL-PSMA, although not statistically significant.

With the expected approval of PSMA RLT prior to chemotherapy, we will be confronted with the dilemma of whether or not a patient would benefit from receiving chemotherapy before PSMA RLT. TheraP demonstrated that PSMA RLT decreased PSA more than chemotherapy but did not show an overall survival (OS) benefit, although it was not powered for OS [6], and it showed that SUVmean is associated with a better treatment response, indicating that PSMA acts as a positive prognostic biomarker, which is consistent with the fact that patients with a higher SUVmean had a greater PSA response to taxane based chemotherapy in our cohort [14]. Additionally, PSMAFore did not show an OS benefit, but did demonstrate an improvement in progression free survival [8]. The decreased SUVmean after chemotherapy suggests that potentially PSMA RLT might have improved efficacy prior to chemotherapy rather than after chemotherapy. Additionally, we observed a more pronounced decrease in SUVmean along with a higher number of cycles in PSA responders compared to non-responders, which may imply that PSMA expression diminishes with an increasing number of chemotherapy cycles. Although in patients with lower uptake, one might consider chemotherapy prior to PSMA RLT.

The trend towards an increased SUVmax and a decreased SUVmean suggests that chemotherapy increases heterogeneity of PSMA uptake within patients. This suggests that in existing disease there is decreased uptake consistent with response resulting in a decreased SUVmean, but at the same time there are regions with progressing lesions that result in higher SUVmax. This mixed treatment response seen on PSMA PET introduces heterogeneity which has been shown to correlate with poorer response to PSMA RLT [15].

The PSA/TL-PSMA ratio demonstrated a positive correlation between baseline and post-treatment PET scans, yet 64% of patients exhibiting a greater than 50% change in this ratio following treatment. This variability highlights differences in response assessment when using PSA alone versus PSMA PET metrics, with nearly 60% of patients having a different classification of response using both metrics. This discrepancy suggests that PSA and PSMA PET capture distinct aspects of tumor biology, and that PSA alone may be insufficient as a biomarker of response. This matches findings by Fanti et al. [16] who emphasized the role of PSMA PET in identifying early treatment failure and enabling prompt adjustments in therapy.

Factors like high tumor burden, low hemoglobin, compromised performance status, and multiple prior treatments impact the efficacy and tolerance of RLT, particularly in taxane-treated patients [17]. Chemotherapy-induced toxicities often lower the threshold for RLT-related adverse events, hampering patient tolerance. In PSMAFore, it was found that the rate of grade 3/4 and serious adverse events was lower in the PSMA RLT arm compared to APRI switch. Moreover, PSMA RLT had discontinuation rates of around 5% and fewer dose adjustments compared to the hormonal treatment arm [8]. This study builds upon these findings by highlighting that taxane chemotherapy is associated with reduced PSMA uptake, even as tumor volume increases. Patients with baseline SUVmean ≥ 10 and those classified as responders demonstrated a greater reduction in PSMA expression on post-chemotherapy scans. This observation further supports the use of PSMA RLT prior to chemotherapy, as it may preserve PSMA expression and enhance the effectiveness of radioligand therapy.

Our study has several limitations. First, this was a retrospective study with a small cohort size. The current study is based on a single-center experience. Additionally, the timing of the PSMA PET and the PSMA PET protocol was not standardized across patients; however, a maximum time window was implemented. Also the SUVmean measurements do not correct for partial volume averaging, which can effect the quantification in small lesions. Similarly, timing of PSA measurements was not standardized across patients and confirmation of PSA responses was not routinely performed. Further research is needed to identify the factors and mechanisms influencing PSMA expression both in vitro and in vivo. Prospective studies are also required to gain a better understanding of PSMA expression following the initiation of taxane chemotherapy, particularly regarding the duration and repeatability of this phenomenon.

## Conclusion

We demonstrate that taxane-based chemotherapy is linked to decreased PSMA uptake, particularly in patients with high baseline SUVmean and responders. These findings suggest that PSMA RLT may be more effective when administered before chemotherapy, as it PSMA expression may be higher with more uniform uptake thereby enhancing radioligand therapy efficacy. However, given the role of PSMA expression as a prognostic factor, the sequence of treatments may influence the efficacy of subsequent therapies, warranting consideration when evaluating the implications of these results. Additionally, the observed heterogeneity in PSMA expression post-chemotherapy, indicated by mixed response patterns on PET scans, may impact subsequent RLT outcomes. Further prospective studies are needed to better understand the optimal sequencing of PSMA RLT and chemotherapy.

## Electronic supplementary material

Below is the link to the electronic supplementary material.

Supplementary Material 1

## Data Availability

The datasets used and/or analysed during the current study are available from the corresponding author on reasonable request.
