# Supplementary material for: Pre- or post-chemotherapy: effect on PSMA uptake
Source: EJNMMI Res. 2025 Apr 7;15:36. doi: 10.1186/s13550-025-01229-3 (PMC11977060; doi:10.1186/s13550-025-01229-3)

**Supplementary Figures**

**Supplemental Figure 1**: Box plots showing the change in post-treatment PSMA PET quantitative parameters: SUVmean, SUVmax, Total lesion PSMA uptake (TL-PSMA) and Total Tumor volume (TTV) for PSA responders and PSA non-responders.


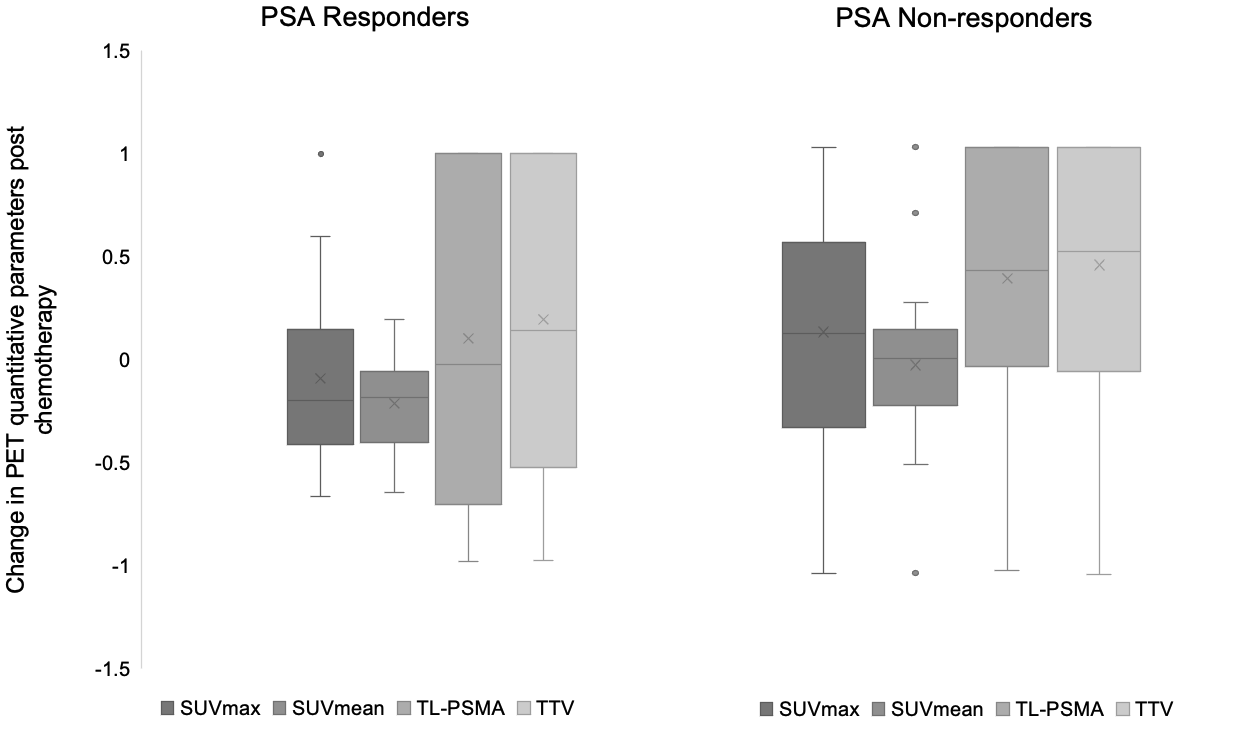


**Supplemental Figure 2**: Scatter plot showing relationship between PSA and TL-PSMA at baseline and at time of post-chemotherapy PET.


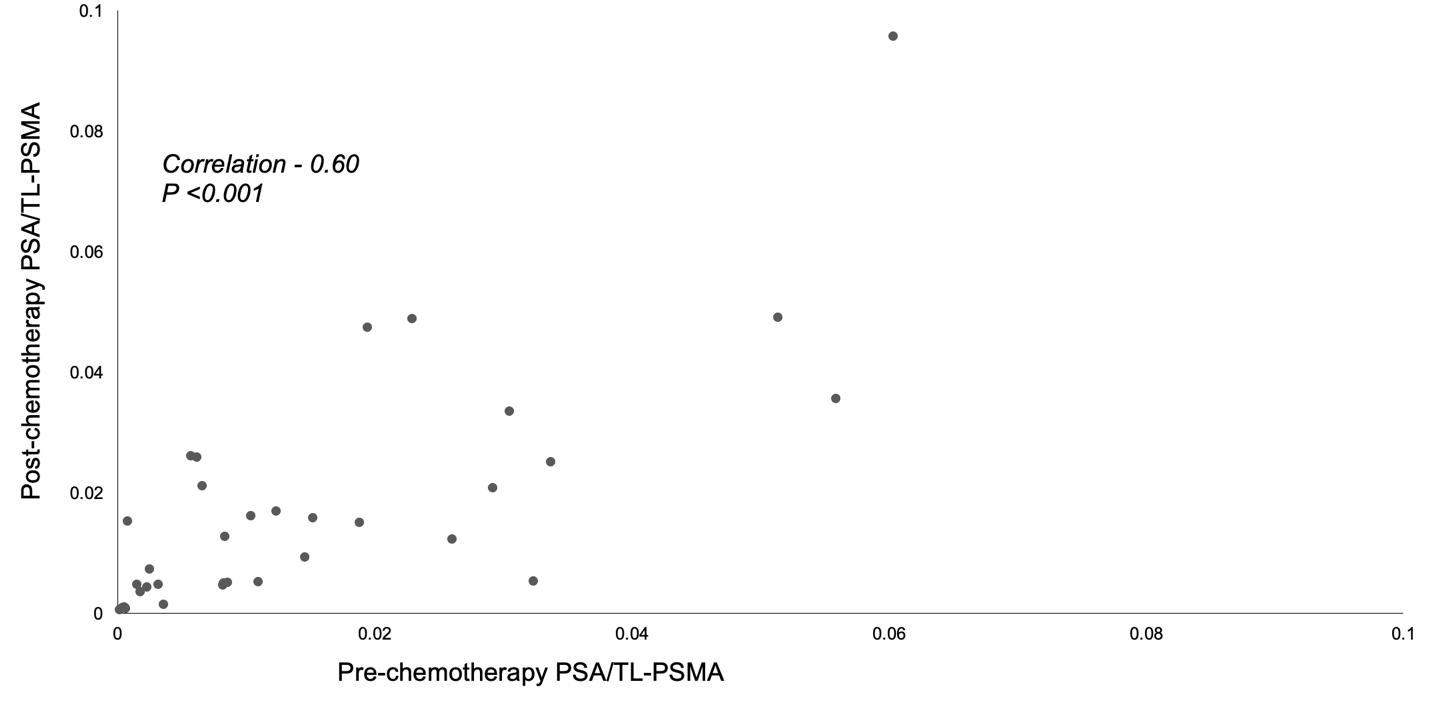

Supplement: Supplementary file 1 — Supplementary Material 1 [file 13550_2025_1229_MOESM1_ESM.docx]
